# Design principles of ion selective nanostructured membranes for the extraction of lithium ions

**DOI:** 10.1038/s41467-019-13648-7

**Published:** 2019-12-19

**Authors:** Amir Razmjou, Mohsen Asadnia, Ehsan Hosseini, Asghar Habibnejad Korayem, Vicki Chen

**Affiliations:** 1 UNESCO Centre for Membrane Science and Technology, School of Chemical Engineering, University of New South Wales, Sydney, NSW 2052 Australia; 2 Department of Biotechnology, Faculty of Biological Science and Technology, University of Isfahan, Isfahan, Iran; 3 School of Engineering, Macquarie University, Sydney, NSW 2109 Australia; 4 School of Civil Engineering, Iran University of Science and Technology, Tehran, Iran; 5 School of Chemical Engineering, University of Queensland, St. Lucia, QLD 4072 Australia

**Keywords:** Chemical engineering, Nanoscale materials

## Abstract

It is predicted that the continuously increasing demand for the energy-critical element of lithium will soon exceed its availability, rendering it a geopolitically significant resource. The present work critically reviews recent reports on Li^+^ selective membranes. Particular emphasis has been placed on the basic principles of the materials’ design for the development of membranes with nanochannels and nanopores with Li^+^ selectivity. Fundamental and practical challenges, as well as prospects for the targeted design of Li^+^ ion-selective membranes are also presented, with the goal of inspiring future critical research efforts in this scientifically and strategically important field.

## Abstract (web summary)

Lithium is in increasing demand for energy storage and is abundant in seawater, but its extraction is challenging due to coexistence with similar ions. Here the authors review recent advances in lithium separation strategies, focusing on the development of nanochannel and nanopore based membranes.

## Introduction

Lithium consumption has been increasing substantially worldwide from 265,000 tons in 2015 (based on Li_2_CO_3_) to an estimated 498,000 tons in 2025 (ref. ^1^). This sharp increase in Li demand is predominantly due to the extensive use of Li-ion batteries (LiBs) or electronic devices. Indeed, over the course of five years from 2010 to 2015, the consumption of LiBs leaped from 4.6 to 7 billion units^1^. Currently, the main sources of Li^+^ supply are brine deposits and lithium ores which are reported to amount to approximately 34 million tons worldwide^2^. While these Li^+^ reserves are sufficient to address the current market demands, the conventional technologies to extract Li^+^ from the resources are either difficult or require high-cost investment^3^, and will struggle to meet future market demands. Furthermore, a major bottleneck is the distribution of conventional resources of Li^+^ around the globe, many in less accessible regions. In comparison, the Li^+^ reserves in seawater are estimated at 230,000 million tons^4^. This unconventional resource of Li^+^ is not limited to a geographic boundary. However, Li^+^–seawater processing is complicated due to the low concentration of Li^+^ (0.1–0.2 ppm^4^) and the coexistence of chemically similar ions such as Na^+^ and K ^+^. Therefore, the development of new processing technologies with enhanced product yields are urgently needed.

The concentration of lithium in brine ranges from 200 to 700 ppm, and can be categorized into various types based on the nature of the salt ions^3^. Although Li^+^ concentration in brine is higher than that of seawater, the rapid depletion of ground resources and its demand projection is the current concern. Innovative brine enrichment processes that can be applied to selectively enrich and extract the Li^+^ from brines are urgently required. The conventional approach to extract Li^+^ from aqua-based resources such as brine and seawater consists of three main stages, namely (i) enrichment (solar evaporation, adsorption, and diffusion dialysis), (ii) purification (solvent extraction, ion exchange, or adsorption), and (iii) Li^+^ precipitation (predominately by adding Na_2_CO_3_). Pankaj et al.^1,3^ recently reviewed the conventional Li^+^ extraction methods, and therefore they are not reviewed here.

Membrane technologies including microfiltration (MF), ultrafiltration (UF), nanofiltration (NF), and reverse osmosis (RO) have been used for water and wastewater treatment including desalination for many years^5^. Membranes can be made from organic and inorganic materials, and their structures vary from the finely porous structures to nonporous. Membranes can separate contaminants such as bacteria and protozoa (1–10 µm), down to gases and ions (0.1–1 nm). They can be classified according to their driving force including concentration difference Δ*C* (forward osmosis (FO), dialysis and pervaporation), potential difference Δ*E* (Electrodialysis), pressure difference Δ*P* (including MF, UF, NF, and RO), and temperature difference Δ*T* (membrane distillation, MD). The most popular materials used for membrane fabrication are polymers, due to their chemical and thermal stabilities, high mechanical strength as well as ability to form into different morphologies such as flat sheets or hollow fibers. For commercial membrane production, the phase-inversion process and interfacial polymerization are the two most widely used preparation techniques^6^.

Among the techniques of the membrane extraction, the nanofiltration processes have been widely used for preconcentration and Li extraction from a lithium-bearing brine. Lithium brines usually contain high Li^+^ concentrations (>5.0 wt. %). Practically, the maximum salt concentration that the membrane processes can achieve is a function of the osmotic pressure and NF membrane selectivity. It is well-known that in NF the combination of Donnan exclusion, steric hindrance, and dielectric exclusion controls the mass transfer^7^. Nanofiltration, having smaller pores (molecular weight cut-off, MWCO, ranging from 0.2 to 1 kDa), is more suited for Li^+^ ion separation purposes. Unfortunately, due to the poor monovalent selectivity of nanofiltration, it can only be used to concentrate Li^+^ by removing all the divalent ions mostly Mg^2+^. It should be pointed out here that the conventional NF processes suffer from severe inorganic fouling and scaling; they cannot efficiently extract Li without a heavy pre-treatment stage, and they require diluting the brine with a large quantity of freshwater. For example, Somrani et al^8^. used negatively charged NF90-2540 membranes for the extraction of Li with Mg/Li mass ratio of 56.76. Although their study showed 100% and 15% rejections for Mg^2+^ and Li^+^, the separation of Li^+^ from Na^+^ was unsatisfactory. In addition, they found that NF90-2540 suffers significantly from fouling (50% reduction in pure water flux). It is also reported that positively charged NF membranes have shown higher Li selectivity than negatively charged ones. According to Donnan effect, the negatively charged NF membranes are suitable for the rejection of anions while they exhibited unsatisfactory separation permanence when treating solutes with positive charges^9^. A variety of positively charged organic molecules such as polyethyleneimine^9^, Ethylenediaminetetraacetic acid (EDTA)^10^, and 1,4-Bis(3-aminopropyl)piperazine (DAPP)^11^ were used to created positively charged NF membranes.

Electrodialysis (ED) using Ion exchange membranes (IEM) such as the well-known Nafion have been used extensively for desalination and ion separations; however, conventional IEMs do not possess Li^+^ selectivity sufficient to meet industry requirements. Nie et al.^12^ showed that ED could be used to effectively separate Li^+^ from brine solution containing a high concentration of Mg^2+^ with a Li^+^ recovery of 95.3%; however, the recovery substantially dropped to around 20% after three hours of ED when K^+^ and Na^+^ were added to the feed. Recently, in a series of reports, Hoshino et al.^13–15^ used organic membranes impregnated with an ionic liquid for Li^+^ separation. However, the poor durability of the ionic liquid membrane was identified as the main issue for stable long-term Li^+^ recovery^16^. Therefore, there is a continuing quest for ion-selective membranes that can selectively separate Li^+^ from similar monovalent ions.

Other membrane techniques such as FO^17^, RO^8^, MD^18^, membrane capacitive deionization^19^, membrane crystallization^20^ and solid electrolyte electrolysis-based technology^21^ have also been used to extract Li^+^ from brine and seawater. Although the techniques exhibited promising Li^+^/multivalent extraction performance, they still cannot selectively separate Li^+^ ions from other monovalent ions.

Rapidly growing knowledge about nanostructured materials (nanopores and nanofluidic channels), recent advances in analytical techniques, and the advent of sophisticated nanofabrication technologies provide us with a great opportunity to strategically design materials. The advanced materials can serve as building blocks for novel membranes with Li^+^ ion selectivity and high permeability. It is well-known that ions behave drastically differently in nano-scale confined environments, as compared with their behavior in bulk solutions^22^. This unexpected and surprising phenomenon in nanochannels and nanopores, along with the special characteristics of Li^+^, serves as an important impetus to design Li^+^ selective membranes.

This review addresses the fundamental concepts relevant to ion transport within nanopores and nanochannels, in addition to discussing the key principles of materials design for the development of membranes with nanochannels and nanopores with Li^+^ selectivity. Also, most recent studies from the literature on the development of such membranes are reviewed. Finally, challenges and prospects for future work in the field have been presented, with the goal of motivating important and timely research that will lead to improved industrially-applicable membrane separation platforms.

## Ion transport in nanochannels

Many reviews have discussed ion transport mechanisms in nanopores and nanochannels, however, it is important to summarize the key concepts herein^23–26^. Briefly, to observe ion selectivity, the chemistry and morphology of the nanochannel need to be carefully adjusted. To make a surface in a nanochannel cation-selective, it should first have fixed negative charge bounds. The space over the charged surface in the nanochannel should then be reduced to nanoscale distance via reducing the nanochannel size, at which the fixed charges begin to show some effect. The distance can be estimated by the evaluation of the Debye length which characterizes the electrical double layer (EDL) that forms at the charged interfaces^24^. Debye length is dependent on the ionic strength of the working buffer solution and under most actual ionic conditions, it is between 1 and 100 nm (e.g., 1 nm for a 0.1 M 1:1 electrolyte). EDL is modeled by a Stern layer and a diffuse layer. The first is the layer of immobilized counter ions (here positively charged) very close to the charged surface of the channel (here with a negative charge), while the counter ions in the latter layer experience the (electrical) attraction of the channel surface. The electrical attraction of the diffuse layer is weaker than the Stern layer, and as a result the counter ions are able to diffuse freely^24^. The potential *ɸ* on the charged surface is a maximum, which reduces to the zeta potential *ƺ* and decays exponentially to zero at a distance from the surface where the concentrations of cations and anions in the bulk are the same. However, when the space over the charged surface decreases such that the double layers overlap, *ɸ* will not decay to zero in the nanochannel, and the counter ions become the dominating charge carriers, leading to the creation of ion-selective nanochannels

When an electrical potential is applied between the two sides of the nanochannel using a pair of electrodes, anions and cations flow toward the positive and negative electrodes respectively. The nanochannel conducts almost exclusively cations.

The reduction in the nanochannel size and electric field could enhance the nanochannel ion conductivity up to several orders of magnitude. Reducing the nanochannel’s dimension below Debye length can have other consequences, such as internal/external ion enrichment, extended polarized layers, surface governed charge transportation, large mobile ion concentration gradients, water stripping, current rectification, and nonlinear ion current circuitry, amongst other phenomena^24^. It should be pointed out that the Debye length is not the only important factor for ion selectivity. It is also affected by other parameters such as the concentration of fixed charge, nanochannel/pore size and morphology, and the intensity of the electrical field.

Ion selectivity can also be explained based on the classical Eisenman sequence^27^ which proposes that there exist only limited selectivity orders of alkaline metal ions. The classical thermodynamic explanation of ions selectivity emphasizes the relative free energy difference of ions in a nanochannel relative to the bulk solution^28^. According to Eisenman, ion selectivity of nanochannels can originate from the energy of the interaction between the ion and a charged binding site within the nanochannel, and the difference between the hydration free energy of the ion^29^. In Eisenman’s theory, the anionic field strength of the binding sites is the critical factor that determines the selectivity sequence of the nanochannel. At the highest anionic field strength, the nanochannel could exhibit a selectivity sequence of Li^+^ > Na^+^ > K^+^ > Rb^+^ > Cs^+^ (known as Sequence XI), while at the lowest anionic field strength the nanochannel could show a reverse selectivity sequence of Li^+^ < Na^+^ < K^+^ < Rb^+^ < Cs^+^ (Sequence I).

One important realization is that ions need to overcome many obstacles (energy barriers) and binding sites (energy wells) as they move through the nanochannel. However, binding and conduction of ions can act as opposing processes; ions need to strip their hydration shells in order to selectively enter the opening of the nanochannel, but they cannot move quickly if they bind too tightly. A way to understand the ion permeation is to consider that ions could jump from one binding site to the next over a series of barriers. It is also important to note that adjusting the ion selectivity of the artificial nanostructured membranes based solely on the dehydration energy and monotonically size-dependency (the dehydration effect at the entrance) will mostly lead to Eisenman Sequence I rather than Sequence XI, as Li^+^ ions have the greatest hydration energy among the alkaline metal ions. Therefore other parameters such as binding sites, nanochannel morphology, the transport speed of the partially dehydrated ions in long nanochannels, cohabiting of multiple ions inside the nanochannel and their interactions, the degree of rigidity of the nanochannel cavity, etc. must be carefully considered. Currently, no general rule has been well-accepted for Li^+^ ion selectivity. The fundamental knowledge and basic principles of artificial nanostructured membranes with Li-ion selectivity are in the embryonic stage and require theoretical and experimental considerations. This is certainly a promising and challenging avenue to pursue. If the pressure difference across the negatively charged nanochannel is used as the driving force, the electrostatic repulsion force of the nanochannel mouth excludes anions while allowing cations to enter.

From a practical point of view, membrane fouling, i.e., inorganic, organic and biofouling, is also important for the effectiveness of extraction of Li^+^ from brine or seawater. Since fouling mitigation has been well-studied and can be controlled by choosing the right pre-treatment process^30–32^, it is not covered here as a contributing factor affecting the Li^+^ separation efficiency of nanofluidic membranes.

Other important environmental factors which can affect the dynamic selective separation of ions such as operating pressure, pH, feed and product concentration, flow velocities, current density and leakage, concentration polarization, and temperature fluctuation, are also important to consider. The effects of the parameters on the separation of Li^+^ from divalent ions (e.g., Mg^2+^) have been studied mainly for nanofiltration and electrodialysis^33^. Generally, it was found that reducing the Mg/Li mass ratio of feed^12^, increasing temperature^34^, high flow velocity (high turbulence and low boundary layer thickness)^35^, higher current density^35^, elevated operating pressure^33^, low pH (4–5)^33^ had a positive effect on the Li^+^ separation. Optimizations of the influential parameters are necessary to maintain the long performance of the membranes. For example, high temperate could reduce the service life of the membrane, or low pH (<3) would cause H^+^ to compete with Li^+^, or high current density increases energy consumption and may lead to the secondary effects such as ion deficit at the membrane-solution interface, water stripping, and concentration polarization. Excessive flow velocity could mechanically damage the membranes and also causes an additional burden on the pumps.

## Effect of nanochannel size

The nanochannel size is considered the most important parameter to control Li^+^ selectivity. In a similar fashion to gas separation in membranes, reducing the size of the nanochannel (*d*_n_) increases the Li^+^ selectivity, but at the expense of permeability. There are a few factors that must be taken into consideration when adjusting the nanochannel size to achieve Li^+^ selectivity. If the inner wall surface of the nanochannel does not possess a negative charge, the nanochannel size must be smaller than the hydration ionic diameter (*d*_H-Li_) of Li^+^ (0.764 nm). It is well-known that for non-charged nanochannels with size *d*_n_ < ~0.764 nm, alkali metal ions must lose part of their hydration layer to enter the channel^36–38^. Dehydration of ions is widely acknowledged as an important factor during the ions transportation in the nanochannel whose size is smaller than the hydrated alkali metal ions. In biological ion filters, ion dehydration plays a crucial role to obtain ion selectivity. Separating of ions based on their ability to dehydrate is feasible as the dehydration-based energy barriers of cations are different when they permeate through nanochannels. Unlike protons that can be transported in the absence of water in hydroxyl functionalized graphene nanochannels^39^ or anhydrous transport of K^+^ ions in biological K^+^-membrane protein ion channels (direct Coulomb knock-on mechnism^40^), to date, there are no reports that synthetic nanochannels that can conduct ions efficiently under anhydrous conditions have been made.

It should be noted that ions require some hydration layer to pass through current synthetic nanochannels; for example, K^+^ ions in dimethyl sulfoxide (DMSO) showed no ion transportation through 0.9 nm GO laminates^41^. The Li^+^ selectivity and conductivity of nanochannels with uncharged walls are discussed further in the following sections.

**Fig. 1:**
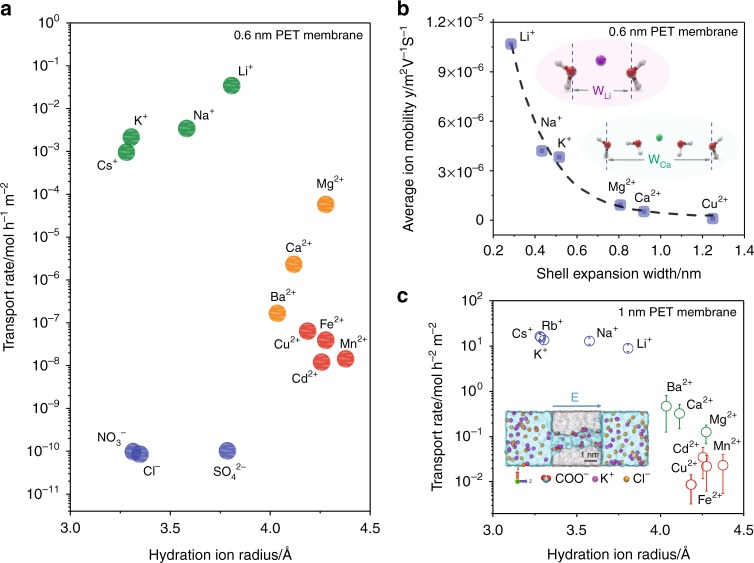
Effect of nanochannel size on Li-ion selectivity and performance. **a** Transport rate vs. hydration ion radius and **b** ion mobility vs. hydration shell width *w* of ions inside 0.6 nm diameter PET membrane. The dashed line in **b** is a guide to the eye. Insets represent partially dehydrated Li^+^ ions having more compact structures, with smaller size of the hydration shell, than Ca^+^ and thus exhibit higher ion mobility and conductivity through the nanochannels. Reprinted with permission from ref. ^42^ Copyright 2019 John Wiley and Sons. **c** Ionic transport rates of cations as a function of hydration radius in 1 nm diameter PET membrane. The inset represents the model structure of the PET nanochannel. Filled circles; brown = CL^−^, purple = K^+^, green and red = COO^−^. Error bars represent the range of measurements of transport rate over the many events (**c**), reprinted from ref. ^43^.

The ionic diameters of hydrated alkali metal ions are between 0.658 and 0.764 nm. Although alkali metal ions have smaller d_H_ than Li^+^, it is reported that the ion mobility of partially dehydrated Li^+^ in the nanochannels could be higher than that of the other alkali metal ions^42^. Further investigations on the ion mobility of different ions within the nanochannels are necessary to draw general conclusions. The effect of the size of the nanochannel with its charged surface on Li-ion selectivity and conductivity is not as critical as for nanochannels with uncharged walls, yet it is still very important. Wen et al.^42^ prepared an ion-selective flexible polyethylene terephthalate (PET) membrane by using swift heavy ions and UV radiation of 12-μm-thick Hostaphan films, showing the order of the transportation followed the trend of Li^+^ > Na^+^ > K^+^ > Cs^+^ »Mg^2+^ > Ca^2+^ > Ba^2+^ (see Fig. 1a). The ions and UV radiation generated many nanochannels of 0.6 nm diameter. The inner channel walls possessed negative charges due to the production of carboxylic acid groups during ion etching in the track core along the penetration path of the swift ions. As can be seen in Fig. 1b, the authors also determine by MD simulation that partially dehydrated Li^+^ have more compact structures with smaller size of the hydration shell than Na^+^ and K^+^ ions, and thus exhibit higher ion mobility and conductivity through the nanochannels. According to the authors’ simulation, the Li^+^, Na^+^, and K^+^ ions and water molecules were transported in a single‐file pattern with the same number of hydrated water molecules (two hydrated water molecules). Since the bare ionic diameter of Li^+^ is smaller than that of Na^+^ and K^+^ ions, it could possess a more compact structure inside the nanochannel.

In further work^43^ from the same group, the nanochannel size of the PET membrane was increased to 1 nm in diameter and the thickness of the membrane was reduced by using 2-μm-thick PET Lumirror films. A substantial increase in the Li-ion transportation rate per unit area was observed from ≈1 × 10^−2^ mol/h m^2^ for the 0.6 nm PET membrane, to >10 mol/h m^2^ for the 1 nm PET membrane, however, the membrane lost its Li-ion selectivity, showing a trend in the transport rate of Li^+^ < Na^+^ < K^+^ < Rb^+^ (Cs^+^) (see Fig. 1c). The poor Li^+^ selectivity arose because the size of the nanochannel was higher than that of the *d*_H-Li_ of Li ions. But the 1 nm PET membrane maintained its Li-ion selectivity with regards to alkali earth metal ions (Li^+^/Mg^2+^ < Li^+^/Ca^2+^ < Li^+^/Ba^2+^) or heavy metal ions, as shown in Fig. 1c. It would appear that increasing *d*_n_ for charged nanochannels with carboxylic groups above the *d*_H-Li_ reduces the Li^+^ to alkali metal ion selectivity of membranes but still shows Li^+^ to alkali earth metal ion selectivity with significantly higher ion permeation rates. In this work, the authors also found that PET membranes with no, or low negative charge density do not show Li-ion selectivity regardless of nanochannel size. (The effect of surface charge density is discussed in Section 4.) The next paragraphs discuss how a nanochannel constructed from different materials can still exhibit Li-ion selectivity even without charge.

**Fig. 2:**
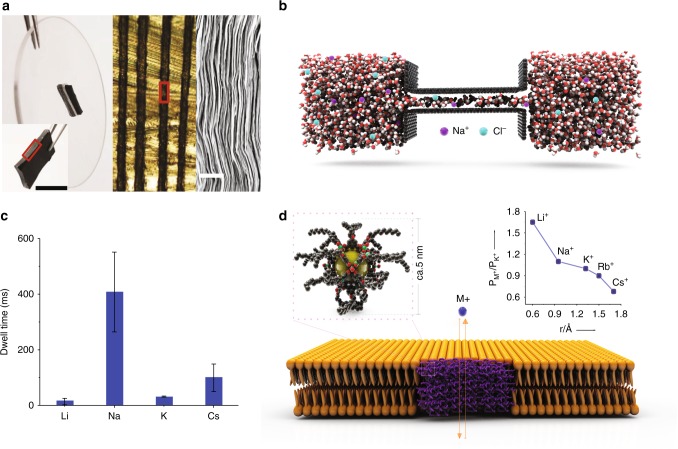
Effect of nanochannel size on Li-ion selectivity and performance. **a** Structure of a graphene oxide (GO) laminar membrane with controlled interlayer spacing. The left image is a photograph of the GO membrane glued into a rectangular slot within a 5 mm diameter plastic disk. The inset represents the photo of the GO membrane before it was placed inside the slot, scale bar: 5 mm. The middle image is the optical micrograph of the cross-sectional area marked by a red rectangle in the left image, presenting 100-μm-thick GO laminates (black) embedded in epoxy marked (light yellow). The right image is the SEM of the region marked in red in middle image, with a scale bar, 1 µm^44^. MD simulation in **b** shows ions and water molecular arrangements within a 0.9 nm graphene slit. For ion transport at least two or three layers of water molecules are necessary when the driving force is a pressure difference, reproduced from ref. ^41^ **c** Dwelling time of monovalent cations in SWNT exhibiting a rapid transport of Li^+^ with the smallest dwelling time following the order of Li^+^ < K^+^ < Cs^+^ < Na^+^(ref. ^48^) (Error bars represent the range of measurements over the many events for SWNT). Panel (**d**) shows the schematic diagram of a synthetic ion channel formed by MOP-18 (5 nm) in lipid bilayers with framework formula of Cu_24_(5‐OC_12_H_25_‐mBDC)_24_ and a large cavity (yellow sphere, diameter 13.8 Å). The inset is the selectivity of monovalent cations (M^+^) with regards to K^+^ as a function of their hydration diameter obtained from reversal potentials (the biomimetic membrane showed Li^+^/ K^+^ selectivity of around 1.7 and ion transport activity in the order of Li^+^ > Na^+^ > K^+^ > Rb^+^ > Cs^+^). Reprinted with permission from ref. ^50^ Copyright 2019 John Wiley and Sons.

As mentioned, when the nanochannel walls are neutral, the effect of nanochannel size on the monovalent ion selectivity becomes critical. Abraham et al.^44^ controlled the interlayer spacing in graphene oxide (GO) laminar membranes between ∼0.64 and 0.98 nm by physically restraining the membrane from swelling. They prepared the GO membranes with a sieve size smaller than the hydration ionic diameter of Li^+^ ions by keeping the membranes under controlled humidity from 0 to 100%. To avoid further dimensional changes in the GO membranes after exposing to water, they embedded the membranes in epoxy (see Fig. 2a). The un-embedded GO membrane soaked in water showed an interlayer spacing of 1.37 ± 0.3 nm. In a pressure-driven membrane process, the authors observed that reducing the spacing systematically in steps from 0.98 to 0.74 nm resulted in a significant exponential reduction (by two orders of magnitude) in the ion permeation rate, whereas the water permeation rate was only reduced linearly. These trends confirmed that ions need to shed their water molecules to enter this narrow channel, and there is a significant energy barrier for partial dehydration of ions to enter the pores. Although the embedded GO membrane showed a higher permeation rate for Li^+^, Na^+^, and K^+^ ions than that of Ca^2+^ and Mg^2+^ ions, no selectivity was observed between Li^+^ and Na^+^ or K^+^. Furthermore, membranes with an interlayer spacing of 0.64 nm exhibited no detectable monovalent ion transported  concentration. Recent molecular dynamics (MD) simulations revealed that GO laminates with a d-spacing of below 0.7 nm do not allow any ion transportation because a monolayer will fill the capillary, but at least two or three layers of water molecules are necessary for transport (see Fig. 2b)^41^. Therefore, for Li^+^ ion extraction using pressure-driven GO base membranes, the lower and upper limit for adjusting the interlayer spacing is between 0.7 and 0.764 nm (*d*_H-Li_). Molecular simulations and modeling have also revealed that chemically modifying the GO nanochannels by introducing charged functional groups does not appreciably change the barrier to ion permeation and ion selectivity^45^. This is not in agreement with the results for other nanostructured materials whose Li^+^ selectivity substantially improved after introducing negative functional groups^46^. Sun et al. also reported that the pressure-driven filtration using GO membranes exhibited poor ion selectivity as the applied pressure could weaken the water-ion interactions in GO nanochannels^47^. It is also not clear how the GO membranes with different interlayer spacing can influence Li^+^ ion selectivity and permeation in an electric-driven filtration system. Therefore, further research is needed to identify the relative importance of nanochannel size and charge.

Choi et al.^48^ considered ion transport through single-walled carbon nanotubes (SWNT) with widths ranging from 0.9 to 2 nm. They observed that up to ~1.6 nm, the flow of K^+^ ions through a nanotube increased steadily, but beyond this diameter, the flow decreased. SWNT also exhibited a rapid transport of Li^+^ with the smallest dwelling time following the order of Li^+^ < K^+^ <Cs^+^ <Na^+^(see Fig. 2c). A similar observation was also made by Lee et al.^49^ Choi et al.^48^ studied ion transportation in isolated single-walled carbon nanotubes (i.e., not in a membrane-like structure), and their findings may help in designing Li^+^ ion-selective membrane with maximum selectivity and throughput; however the need for alignment of SWNTs to construct a membrane-like morphology with high Li^+^/K^+^ selectivity would present a challenge.

**Fig. 3:**
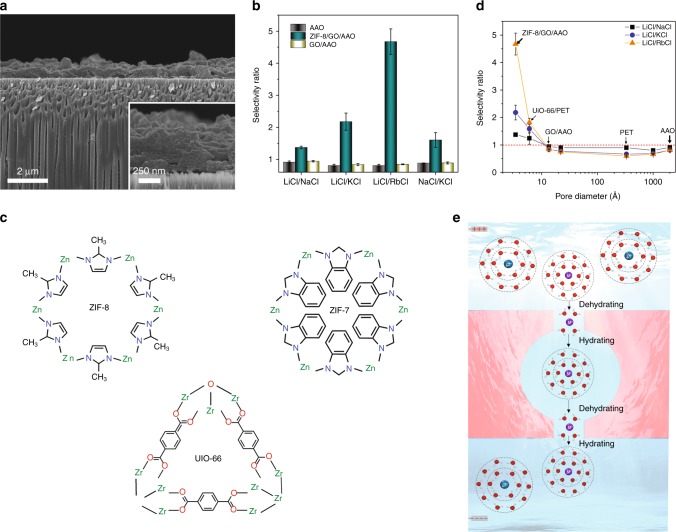
Metal–organic frameworks as promising building blocks of Li-ion selective membrane. **a** The SEM image of ~446-nm-thick ZIF-8/GO layer grown on AAO support showing Li^+^/ K^+^ selectivity of 1.37. The inset shows that the layer is densely grown on the top of the AAO support. **b** Alkali metal ion selectivity of AAO, ZIF-8/GO/AAO, and GO/AAO membranes. Error bars represent the range of measurements of selectivity over the many events. **c** The molecular structures of ZIF-8 with ~3.4 Å window, ZIF-7 with ~2.9 Å window, and UiO-66 with ~6.0 Å window. **d** Ion selectivities as a function of the pore window diameter of different MOFs and of the pore diameter of nanoporous membranes. The figure shows that at the subnanometer scale, the alkali metal ion selectivity of the MOF membranes decreases with increasing window diameter (no alkali metal ion selectivity was observed for membranes with pore diameters >1 nm). **e** Li^+^ ions passing through sub-nanometer ZIF-8 pore experience multiple dehydration–hydration processes (spheres: blue = Cl^−^, purple = monovalent cations that are surrounded by H_2_O molecules. “+” sign is Cathode while “–” sign is Anode), reproduced from ref. ^51^.

Another class of nanostructured hollow materials suitable for use in Li^+^ ion separation is Covalent/Metal^−^Organic Frameworks (C/MOFs) with a narrow distribution of pore sizes. Jung et al.^50^ developed a biomimetic membrane by incorporating MOP-18 into lipid bilayers as shown in the top image of Fig. 2d. MOP-18 with an overall size of 5 nm has a cavity 0.138 nm in diameter with eight triangular (0.38 nm) and six square (0.66 nm) windows. As shown in Fig. 2d, the biomimetic membrane showed Li^+^/K^+^ selectivity of around 1.7 and ion transport activity in the order of Li^+^ > Na^+^ > K^+^ > Rb^+^ > Cs^+^. Although the biomimetic membrane showed Li-ion selectivity, its poor physical and chemical stabilities were not found to meet industry requirements. In an extension of the work on MOF membranes, Zhang et al. showed that a suitable MOF with angstrom-sized windows and nanometer-sized cavities could exhibit excellent Li^+^ selectivity under an electric field^51^. They created a layer of an ultrathin MOF (ZIF-8) membrane, prepared by a nanoporous GO-assisted interfacial growth method on solid-state anodic aluminum oxide (AAO) support (Fig. 3a). Importantly, AAO or GO/AAO membranes did not show Li-ion selectivity without the ZIF-8 top layer (Fig. 3b). The authors also observed that the use of ZIF-7 (middle image of Fig. 3c) with a window size of 0.29 nm resulted in no Li-ion conductivity and selectivity. When UiO-66 (bottom image of Fig. 3c) with a large window size of 0.6 nm was chosen, it showed alkali ion conductivity but poor Li^+^ selectivity. Surprisingly however, Li-ion selectivity (Li^+^/K^+^ selectivity of around 1.37) was observed when ZIF-8 (top image of Fig. 3c) with a window size of 0.34 nm larger than that of ZIF-7, but smaller than that in UiO-66, was examined (see Fig. 3b, d). Since the ZIF-8 windows are neutral and do not possess functional groups with specific ion binding properties, the ion selectivity was attributed to the sub-angstrom differences in partially dehydrated ionic diameters of alkali metal ions. The authors also claimed that when Li^+^ ions are passing through the sub-nanometer ZIF-8 pores, they experience multiple dehydration-hydration processes (see Fig. 3e) as the ions undergo a dehydration process upon entering the ZIF-8 window and a hydration process when they exit the window. This higher ionic mobility of Li^+^ after losing its first hydration layer inside the nanochannel is known as a possible reason for dehydration-assisted Li-ion selectivity within the narrow confines of nanochannels with no charge on the surface and *d*_n_ < *d*_H-Li_. Zhang et al^51^. concluded that the Li^+^ ion-selective mechanism in ZIF-8 pores is attributed to (i) the partial dehydration of ions, (ii) weak interactions between confined ions, water molecules and MOF frameworks, and (iii) the size exclusion of the dehydrated ions. Ionic dehydration is controlled by the size confinement effect while the interactions between confined ions, water molecules and MOF frameworks are mainly governed by van der Waals forces and Columbic interactions.

**Fig. 4:**
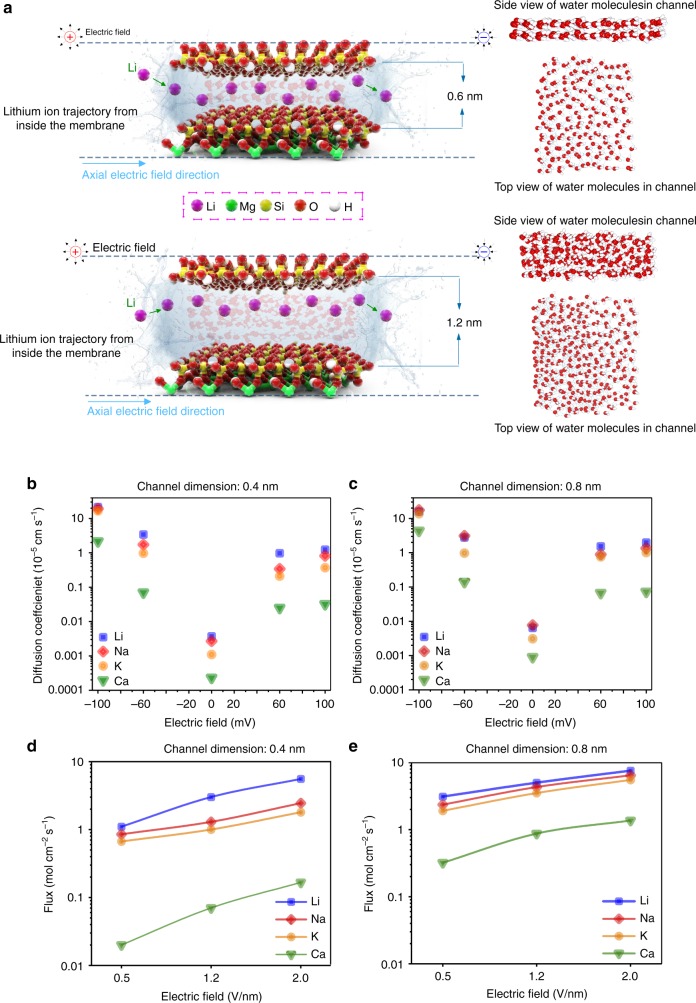
Effect of nanochannel size on Li-ion transport mechanism. The left two images in **a** show Li^+^ ions transport behavior and water molecules orientation inside 0.6 and 1.2 nm vermiculite nanochannels respectively, illustrating how the Li^+^ ions jump between the surfaces of the nanochannel in a zig-zag fashion when the interlayer distance is below 1nm^52^. The corresponding two right images exhibit the effect of reducing the nanochannel size to angstrom scale on the water molecular arrangements from side and top view, revealing that the water density and number of layers inside the nanochannel increases upon increasing channel dimension from 4 to 12 Å. **b**–**e** MD simulation findings on the effect of change in electric driving force and inter-surface distance of 2D vermiculite membrane on the diffusion coefficient of the cations (**b**, **c**) and their ionic flux (**d**, **e**); solid lines are guides to the eye^52^.

We recently studied Li-ion transport within nanoclay (vermiculite) membranes with sub-nanometer 2D channels using experimental and MD simulations^52^. The MD simulation revealed that the water density and number of layers inside the nanochannel increases upon increasing channel dimension from 4 to 12 Å (see Fig. 4a). This could lead to higher ion mobility, as the reduction in water density can facilitate Li^+^ ion movement. Another interesting finding was that Li^+^ ion transportation behavior is a function of the nanochannels dimension. As shown in Fig. 4a, for nanochannels below ~1 nm in size, the Li^+^ ions conduct via the two-surface charge-governed transport mechanism by jumping from one of the nanochannel walls to the opposite wall while moving in the direction of the electric field. However, for channels above 1 nm, the Li^+^ ions moved via one-surface charge-governed transport mechanism. This change in conduction behavior has been attributed to the phenomenon of spontaneous symmetry breaking of charge-regulated surfaces^52,53^. We also reported that the effect of reduction in angstrom size nanochannel dimension from 0.8 to 0.4 nm on the diffusion coefficient and Li-ion flux was significant (see Fig. 4b, c). It was noted there was a trade-off between ion permeability and selectivity as the size reduces.

Another important factor that has yet to be investigated is the adsorption effect, especially between Li^+^ ions and the inner wall surface of uncharged nanochannels and membranes in the presence of multivalent ions that usually exist inside brine and seawater.

## Effect of nanochannel surface charge

Although the nanochannel size and partial dehydration of Li^+^ ions are critical for their entry into the channels, the behavior of Li ions inside the channels can be controlled by manipulating the surface charges. Li-ion selective nanochannels should have negative charge density to exclude co-ions with negative charges, e.g., Cl^−^, and to attract Li^+^ ions with a positive charge. This negative charge density can be introduced through functional groups on the inner surface of the nanochannels. The number of functional groups (FGs) per nm should be carefully adjusted, as having a high number of FGs/nm increases the activation energy required for Li^+^ ions to hop from one site to the next one. By comparison, a low number of FGs/nm does not meet the requirement of surface charge-governed transportation of ions and the Grotthuss mechanism^22^.

**Fig. 5:**
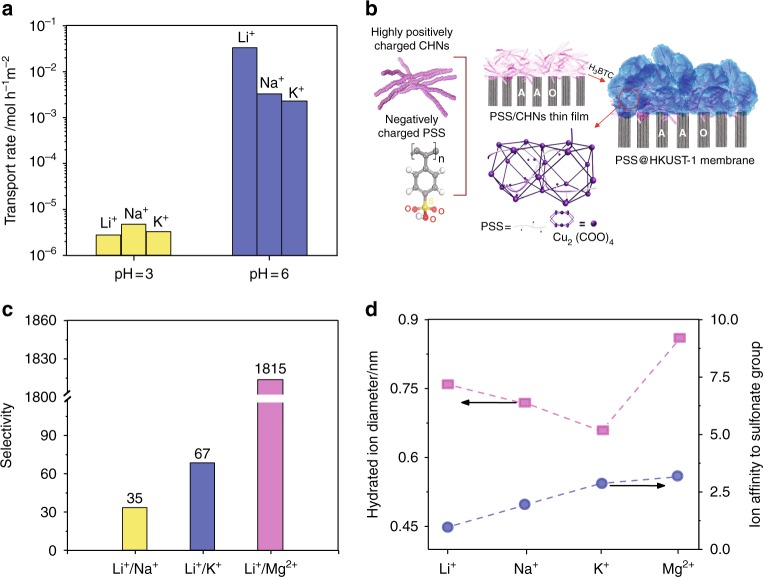
Effect of nanochannels surface charge on Li-ion selectivity and performance. **a** Ionic transport rates of monovalent cations as a function of pH in 0.6 nm diameter PET membrane. The bar chart presents that reducing the pH (protonation) can mitigate the negative charge density of carboxylic acid groups in the nanochannels resulting in a significant drop in the ion transportation rate and thus Li^+^ selectivity, Reprinted with permission from ref. ^42^ Copyright 2019 John Wiley and Sons. **b** The preparation procedures of PSS-threaded HKUST-1 membranes via a solid confinement conversion process from a thin film of copper hydroxide nanostrands (CHNs) that are coated with negatively charged polystyrene sulfonate (PSS) on AAO. **c** The binary ion selectivities of PSS-threaded HKUST-1 (0.5 M LiCl and 0.5 M KCl and MgCl_2_ electrolytes, dashed lines are guids to the eye). The high Li^+^ affinity of sulfonate groups (about four sulfonate groups/nm) to Na^+^ and K^+^ was used to develop a high-performance Li-ion selective membrane. **d** Hydration diameters and ion affinities to sulfonated group related to that of Li^+^, Na^+^, K^+^, and Mg^2+^. Reproduced with permission from ref. ^46^ Copyright 2019 John Wiley and Sons.

Wen et al.^42^ reduced the negative charge density of carboxylic acid groups in 0.6 nm PET nanochannels by reducing the pH (protonation) of the solution from 6 to 3 and observed a significant drop in the ion transportation rate per unit area and Li^+^ selectivity (see Fig. 5a). It would appear that the negative charge groups of the carboxylic acids not only exclude the anions, but also control the passive and time loading (time elapsed between two ions sequentially entering the nanopore) of ions^51^. However, it is still not clear how the distribution of charged carboxylate functionalities and their arrangement inside the channel can alter the Li-ion selectivity and transportation behavior.

Contrary to the relatively limited work on carboxylic acid functional groups, ample information is available regarding the ion transportation mechanism within decorated confined areas with sulfonate groups, owing to Nafion and its applications^54^. It is well-known that the affinity of sulfonate groups for alkaline and alkaline-earth metal ions is different. This affinity difference was used to develop Nafion membranes for fast ion transportation but with very poor ion selectivity, mainly due to a lack of sufficient control over the nano spaces and the existence of many irregular free-spaces as well as ion paths with wide size distributions^55^.

Guo et al.^46^ took advantage of the sub-nanometer (0.9 nm) window size of the HKUST-1 MOF and the high Li^+^ affinity of sulfonate groups of linear polymer polystyrene sulfonate (PSS) (about four sulfonate groups/nm) to construct a high-performance Li-ion selective membrane, see Fig. 5b. Their membrane, which consisted of a PSS-threaded HKUST-1 layer on a solid-state AAO support, exhibited a Li-ion conductivity of 5.53 × 10^−4^ S/cm at 25 °C, an ion transportation rate per unit area of 6.75 mol/h m^2^, and binary ion selectivities of 35, 67, and 1815, for Li^+^/Na^+^, Li^+^/K^+^, and Li^+^/Mg^2+^, respectively. It is very interesting that the incorporation of the sulfonate groups within the HKUST-1 layer with a pore aperture of 0.9 nm (i.e., greater than the sizes of *d*_H-Li_), not only induces fast Li-ion transportation, but also provides the membrane with the high selectivity of Li^+^ over Na^+^, K^+^, and Mg^2+^ (see Fig. 5c).

In contrast to the sulfonated HKUST-1 nanochannels, increasing the pore size of carboxylated nanochannels of PET membranes (from 0.60 to 1 nm) to above *d*_H-Li_, resulted in the loss of Li-ion selectivity over alkali metal ions (see Fig. 1a, c). According to Guo et al.^46^, the high Li-ion selectivity of PSS-threaded HKUST-1 membranes is attributed to the different affinity for Li-ions and other mono or divalent cations. As can be seen in Fig. 5d, Li^+^ has the lowest binding affinity (normalized to Li^+^) with sulfonate groups compared with Na^+^, K^+^, and Mg^2+^. The lower binding affinity results in a more difficult condensation of the cation–sulfonate pairs, and also enables easier dissociation for fast transportation within the nanochannel. Thus, the low Li^+^ bonding affinity and activation energy (0.21 eV) suggests that Li^+^ ion transport through the PSS@HKUST-1 membranes is via a Grotthuss-like mechanism, where the ions hop from one sulfonate group to the next.

**Fig. 6:**
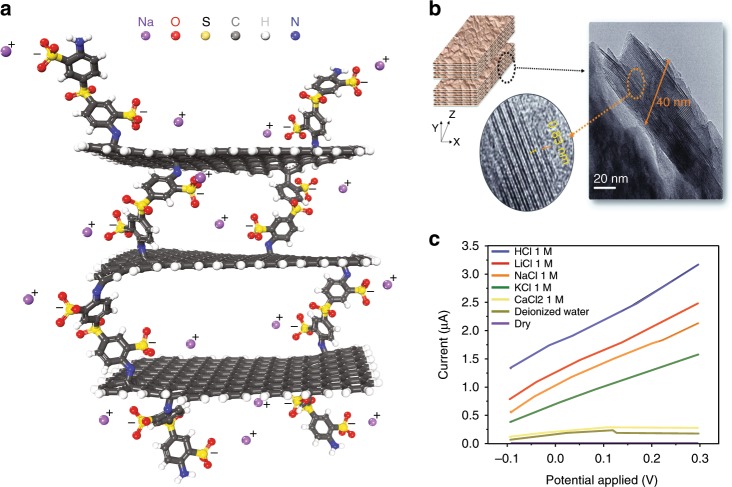
Effect of nanochannel surface charge on Li-ion selectivity and performance. **a** Symmetrical multilayer architecture of rGO modified by grafting of sulfonated 4,4′-diaminodiphenyl sulfone (rGO-SDDS-rGO). The prepared rGO-SDDS-rGO membrane with an average interlayer spacing of ≈0.48 nm showed Li^+^/multivalent cation selectivity (e.g., 5.27 (Li^+^/Mg^2+^) and 4.72 (Li^+^/Ca^2+^)) whereas it exhibited poor Li^+^/Na^+^ selectivity of 0.84 (the permselectivity of Na^+^/Li^+^ is 1.19). Reproduced with permission from ref. ^56^ Copyright 2019 John Wiley and Sons. **b** 3D model and TEM images (scale bar is 20 nm) of Li^+^-selective vermiculite membrane. The reassembly of exfoliated vermiculite layers resulted in a Li^+^ selective membrane with 0.43 nm 2D nanofluidic channels. The membrane exhibited a high Li^+^ conductivity with surface charge-governed ion conductivity in the order of Li^+^ > Na^+^ > K^+^, and with selectivity ratios of 1.26 for Li^+^/Na^+^, 1.59 for Li^+^/K^+^, and 1.36 for Na^+^/K^+^. The graph in **c** shows *I*–*V* curves of different electrolytic solutions indicating selective transportation of monovalent cations inside the vermiculite membrane, reproduced from refs. ^52,62^.

The addition of sulfonate groups into nano confined space is not always successful, and it seems that the morphology and intrinsic nature of the used material are also important contributing factors that need to be carefully considered. Zhao et al.^56^ tried to introduce sulfonate groups between graphene sheets by grafting the sheets with sulfonated 4,4′-diaminodiphenyl sulfone (SDDS), see Fig. 6a. Although the prepared rGO-SDDS-rGO membrane with an average interlayer spacing of ≈0.48 nm showed Li^+^/multivalent cation selectivity (e.g., 5.27 (Li^+^/Mg^2+^), and 4.72 (Li^+^/Ca^2+^)), it exhibited poor Li^+^/Na^+^ selectivity of 0.84 (the permselectivitys of Na^+^/Li^+^ is 1.19). For 0.48 nm of rGO-SDDS-rGO, the presence of cracks or pinholes in GO thin films played a critical role in ion transportation, however Zhao et al.^56^ did not investigate this observation. In a comment on the work of Zhao et al.^56^, Amir Razmjou^57^ tried to explain the importance of morphological defects on the Li^+^ ion separation performance.

As mentioned previously, MD simulations revealed that chemically modifying the GO nanochannels by introducing charged functional groups, e.g., -OH, could not appreciably change the barrier to ion permeation and ion selectivity^45^. Therefore, more work is needed to clarify why the current surface charge modification of GO did not improve the Li-ion selectivity of GO-based membranes. Also, novel synthetic approaches for producing charged GO-based membranes is required to boost the Li-ion transportation. It is reported that GO is nearly impermeable to water at highly acidic or basic conditions (pH <2 or >11) as well as high salt concentrations (above 100 mmol/L NaCl)^58^. The long-term stability and physically integration of GO, especially under basic conditions, is still under question^59,60^. Lastly, thermal deoxygenation of GO at a relatively low temperature is a further issue that needs to be addressed^61^.

Recently self-assembled Vermiculite-based 2D nanofluidic channels with 1.41 nm interlayer spacing and 1.4 m C/m^2^ behavior negative charge density exhibited promising surface charge-governed proton conductivity (2.6 × 10^−2^ S cm^−1^ at pH of 1)^22^. We recently showed that the reassembly of exfoliated vermiculite layers resulted in a Li^+^ selective membrane with 0.43 nm 2D nanofluidic channels (see Fig. 6b)^52,62^. The membrane exhibited a high Li^+^ conductivity (see Fig. 6c) with surface charge-governed ion conductivity in the order of Li^+^ > Na^+^ > K^+^, and with selectivity ratios of 1.26 for Li^+^/Na^+^, 1.59 for Li^+^/K^+^, and 1.36 for Na^+^/K^+^. 2D nanoclay based membranes have significant advantages compared with GO and other two-dimensional structures: they possess extraordinary chemical and thermal stability and are more readily available. In comparison with the exfoliation procedure for graphene and other 2D materials, 2D nanoclays can be exfoliated easily in aqueous solution via thermal heat treatment and ionic exchange. As mentioned before, the lack of sufficient chemical and thermal stability is the Achilles’ heel of graphene and other 2D materials^22^. The self-assembled Vermiculite membranes suffer from brittleness and mechanical instability, and they need to be sandwiched between PDMS, increasing their footprint and rendering scale-up difficult.

A 2D negatively charged Ti_3_C_2_T_x_ MXene membrane with 6.4Å interlayer spacing in wet state exhibited charge- and size-selective ion sieving^63^. The membrane exhibited high selectivity toward single-, double-, and triple-charged metal cations in the order of Na^+^ > Li^+^ > K^+^ > Ca^2+^ > Ni^2+^ > Mg^2+^ > Al^3+^. The permeation rate of Na^+^ (1.53 mol/h^1^/m^2^) was found to be higher than Li^+^ (1.40 mol/h^1^/m^2^), which is not suitable for Li-ion extraction from brine or seawater.

## Effect of nanochannel morphology

**Fig. 7:**
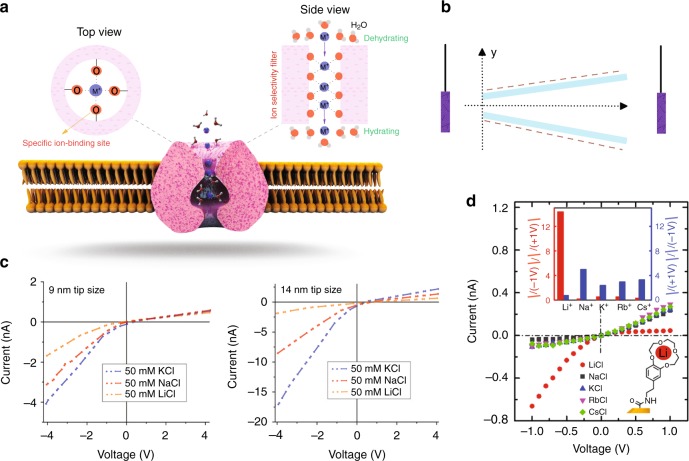
Effect of nanochannel morphology on Li-ion selectivity and performance. Schematic in **a** represents a biological ion channel with an angstrom-sized asymmetric ion selectivity filter and a nanometer-sized cavity for fast ion transport (Spheres: orange = oxygen, blue = monovalent cations that are surrounded by H_2_O molecules before entering and exiting the nanochannel). In the biological protein nanochannels, ions have a chance to gradually lose their hydration shells as they move into the asymmetric ion filter. **b** A asymmetric nanochannel with ion rectification property (lines: purple = electrodes, light blue = nanochannel walls, dashed red = negative charge). **c** *I*–*V* curves of single conically shaped PET nanopores with a tip diameter of 9 and 14 nm. Li^+^ ions showed the lowest rectification and current, with the effect on the *I*–*V* curves becoming less significant when the tip size diameter increased from 9 to 14 nm^70^. **d** The characteristics *I*–*V* curve of PET conically shaped nanopores modified with Li^+^ selective receptor moieties (aminoethyl-benzo-12-crown-4 (BC12C4−NH2), which were exposed to different alkali metal solutions at a concentration of 100 mM and pH of 6.5 (the inset demonstrates the rectification ratio vs. cations at 1 V), Reproduced with permission from^71^ Copyright (2019) American Chemical Society.

The morphology of nanochannels can also affect the Li-ion transportation rate and selectivity. Bare Li^+^ ion size is around 0.120 nm and is smaller than Na^+^ (0.190 nm) and K^+^ (0.266 nm). Theoretically, a nanochannel size can be reduced such that only Li^+^ ions can permeate, exclusively based on size exclusion mechanism. However, practically, ion permeation is sharply suppressed by a factor of 15 if the hydration layer of ions exceeds the nanochannel size by 50%^64^. Currently, there are no reports showing that Li^+^ ions can be transported within a nanochannel with a size smaller than the bare diameter of Na^+^ (0.190 nm). Even if one can make such a small “symmetric” nanochannel, it may not be practically useful because of intrinsically associated issues such as large free energy barriers and dehydration energy for the permeation of ions into the nano capillaries, exit-entry effects, and low permeation rate. However, biological “asymmetric” ion filters have found a way to selectively transport the fully dehydrated angstrom size ions^65,66^. In the biological protein nanochannels, ions have a chance to gradually lose their hydration shells as they move into the asymmetric ion filter, whereas in the synthetic symmetric nanochannels, ions need to immediately lose most of their outer hydration shell and overcome a large hydration energy barrier to enter the nanochannel. For example, Na^+^ channels (Fig. 7a) consist of angstrom-sized (3 to 5 Å) ion selectivity filters and nanometer-sized cavities (1.2 nm). They also exhibit a Na^+^/K^+^ selectivity of 10 to 30, with a selectivity sequence of Li^+^ > Na^+^ > K^+^ > Rb^+^^51^. The Na^+^ protein channels are naturally designed by the assembly of amino acids which possess a series of free carbonyl oxygen atoms (C=O) and functional amine and carboxyl groups. Since the size of the channel is smaller than the *d*_H_, ions should strip their water shell when they bind into the selectivity filters, and subsequently be rehydrated by water molecules as they progress into the large cavity of the ion channel. Based on this concept, Zhang et al.^51^ prepared a hierarchical non-charged nanochannel of ZIF-8 where its small windows and large cavity caused Li ions to experience multiple dehydration-hydration processes and higher selectivity over other alkali metal ions (see Fig. 3). It is worth mentioning that bioinspired smart asymmetric nanochannel membranes and control of alkali metal ions across nanochannels were recently reviewed^67,68^ and thus were not covered here.

For nanochannels with charged surfaces, it is well-known that ion conductivity can be influenced by a phenomenon known as breaking the symmetry of nanopore/nanochannels, for instance, by utilizing non-cylindrical channels, that leads to asymmetric ion transportation, asymmetric current–voltage (*I*–*V*) curves, and ion current rectification^65,67,68^. As shown in Fig. 7b, the channel tip at x, z = 0 is small enough to match the thickness of the electrical double layer, and there is an excess negative charge on the inner walls with asymmetric ions interactions at the two entrances^69^. The asymmetric interaction between ions and the inner channel walls creates the potential internal distribution in the nanochannel. The nanochannel is low in ion current and is in a non-conductive state when the cathode is on the tip side. An electrostatic trap is formed as anions enter the large entrance while they cannot exit the tip (positive external voltages). However, the nanochannel shows high ion currents with no trap when the anode is on the tip side; the negative charge of the inner surface rejects the anion (negative external voltages). It has been reported that the geometric characteristics of the tip, the length of the pore, and the surface charge density of the nanochannel wall can alter the current–voltage (*I*–*V*) curves^70^. It was also observed that the rectification properties of ionic systems could be significantly improved by introducing surface charge patterns inside the pore^26^. Gamble et al.^70^ studied the effect on ion current rectification of changing the tip diameter of single PET conically shaped nanopores from 3 to 25 nm, as measured in KCl, NaCl, and LiCl. As can be seen in Fig. 7c, Li^+^ ions showed the lowest rectification and current, with the effect on the *I*–*V* curves becoming less significant when the tip size diameter increased from 9 to 14 nm. However, when Ali et al.^71^ functionalised the inner surface of the single PET conically shaped nanopores with Li^+^ selective receptor moieties (aminoethyl-benzo-12-crown-4 (BC12C4−NH_2_)), the changes in the *I*–*V* curves became substantial (see Fig. 7d). This is due to the generation of positively charged B12C4−Li^+^ complexes on the pore surface, as evidenced by the clear difference observed between the rectification ratio for Li^+^ and the other cations (see inset image Fig. 7d). The modified nanopore also showed ion current and rectification only for lithium chloride with high selectivity. It seems that the architecture of the nanochannel geometry and charge density is critical for designing Li-ion selective membranes with high throughput, which has not yet been systemically investigated.

## Effect of the driving force

Similar to ion-exchange membranes, nanochannel structures also exhibited a nonlinear current-voltage curve where two ohmic regions are connected through a limiting region, due to a phenomenon that is known as the “ion-enrichment and ion-depletion effect” of nanochannels^72^. Increasing the applied potential and power density of the electrical field (voltage) beyond a certain voltage between the two sides of nanochannel causes the depletion of ions on one side and their enrichment on the other side, resulting in polarization concentration and nonlinearity of the *I*–*V* curve. Increasing the applied potentials above the limiting current again revealed linearity in the *I*–*V* curve associated with the over limiting region. The mechanism by which the over limiting region occurs has been attributed to many possible causes, including electroconvection, water stripping, natural convection, or changes in ion selectivity^73–77^. The current operating limitation (*V*_lim_) should be considered as one of the restrictions for designing Li-ion selective membrane-based systems. Although recently reported nanochannels exhibited promising Li^+^ selectivity and permeability, nonlinearity in their *I*–*V* curve with regards to Li-ion transportation has yet to be studied. Also, the effect of change in applying pressure as the driving force has not been systematically investigated.

**Fig. 8:**
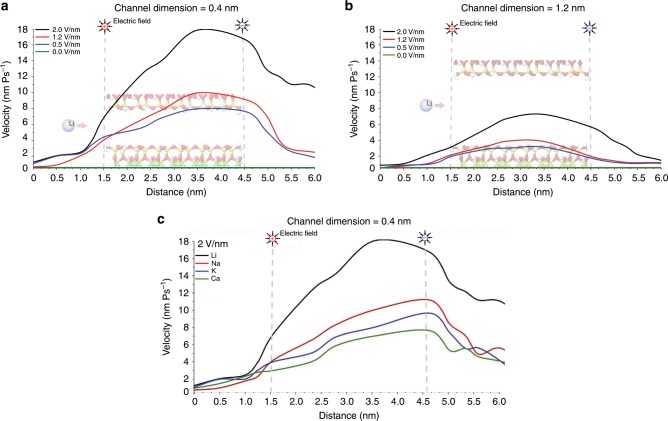
Effect of driving force on Li-ion selectivity and transport behavior. Effect of the power density of electrical field on the velocity of Li^+^ ions inside a 0.4 nm (**a**) and 1.2 nm (**b**) vermiculite nanochannel. Underlying nanochannels representations in **a**, **b** are 3 nm (length) vermiculite nanochannels. The molecular structures represent the layered magnesium aluminosilicate compounds of the nanochannel. Nanochannel entrance and exit points are set at 1.5 and 4.5 nm on the *x*-axis (see Fig. 4a for atom colors). **c** A comparison between the velocity of Li^+^ and that of Na^+^, K^+^, and Ca^2+^ ions in 0.4 nm vermiculite nanochannel^52^. Li^+^ ions could exhibit acceleration-deceleration behavior inside the nanochannel as a function of nanochannel length. The ions exhibited an almost linear velocity gradient and acceleration at the entry to the nanochannel, followed by sudden deceleration. Among Li^+^, Na^+^, K^+^, and Ca^2+^, Li^+^ ions showed the highest acceleration inside the nanochannel, which is the main cause of higher Li^+^ ion flux and consequent selectivity of the membrane.

Using MD simulation, we recently demonstrated^52^ that increasing the electric field power density enhances the diffusion coefficient and ion flux of cations in the vermiculite membrane with 0.4 and 0.8 nm interlayer spacing (see Fig. 4b–e). As can be seen in Fig. 4e, the Li^+^ ionic flux is slightly higher than that of Na^+^, and K^+^ ions for 0.8 nm nanochannel and increased almost linearly. However, Li-ion selectivity (Li^+^-Flux **/** K^+^-Flux) as a function of the electric field was unchanged. Surprisingly, Li^+^ ion selectivity vs. electric field is not constant when the nanochannel size is reduced to 0.4 nm (Fig. 4d). Another important finding (illustrated in Fig. 8) is that Li^+^ ions could exhibit acceleration-deceleration behavior inside the nanochannel as a function of nanochannel length. The ions exhibited an almost linear velocity gradient and acceleration at the entry to the nanochannel, followed by a sudden deceleration. The nanochannel size can also drastically change the acceleration-deceleration behavior of the ions (see Fig. 8a, b). The differing velocity profiles of ions inside the nanocapillary are attributed to water fluidity and density, and a phenomenon known as the “ion-enrichment and ion-depletion effect” of nanochannels^72^. Among Li^+^, Na^+^, K^+^, and Ca^2+^, Li^+^ ions showed the highest acceleration inside the nanochannel (see Fig. 8c), which may be the main cause of higher Li^+^ ion flux and consequent selectivity of the membrane.

## Challenges and perspectives for Li^+^ ion-selective membranes

This section presents a brief overview of the challenges for Li^+^ ion-selective membranes from both fundamental and practical points of view. Although recently reported Li^+^ selective membranes have exhibited promising selectivity, their low permeability makes their practical application limited. Table 1 summarizes Li^+^ ion-selective membranes with regards to the materials used and their nanochannel dimensions, length, and charge on the selectivity and permeation rate.

**Table 1:** Comparison of nanostructured based Li^+^ ions selective membranes that are reported in literature.

Membranes	Ion channel dimension (nm)	Charge density	Li+ selectivity		Li^+^ transportation rate	Length (µm)	Ref.
			Li^+^/Na^+^	Li^+^/K^+^			
UiO-66	~0.6	Neutral	1.24	1.58	—	12	^51^
ZIF-8	~0.34	Neutral	1.37	2.18	~10^5^ ions/s^1^ per single pore	0.45	^51^
ZIF-7	~0.29	Neutral	—	—	—	—	^51^
MOP-18	~0.66	Neutral	—	1.7	—	—	^50^
Sulfonated HKUST-1	~0.9	Negative	35	67	6.75 mol/h m^2^ at 400 mV	8	^46^
PET	~0.6	Negative	10.46^a^	16.00 ^a^	~0.36 mol/h m^2^	12	^42^
PET	~1.0	Negative	0.81^a^	0.77 ^a^	~10 mol/h m^2^	2	^43^
PET	~2.2	Negative	0.9	0.77	—	12	^51^
GO	<0.7	Neutral	—	—	—	—	^41,44^
GO	~0.98	Neutral	0.77^a^	0.83^a^	0.005 mol/h m^2^	100	^44^
GO	~1.35	Neutral	0.93	0.84	—	10	^51^
Sulfonated-rGO	~0.48	Negative	0.84	—	—	—	^56^
Vermiculite	0.43	Negative	1.26	1.59	5 mol/cm^2^ s	—	^52^
MXene	0.64	Negative	0.91	1.5	1.40 mol/h m^2^	—	^63^

^a^The selectivity is calculated based on the transportation rate ratios

Research and development into Li^+^ ion-selective membranes are still in their infancy. Although nanofabrication technologies are being progressed that could move the state-of-the-art in ion-selective membrane processes forward, there are several challenges that need to be addressed before it can be considered mainstream. It seems there are three major challenges for Li^+^ ion-selective membrane: (i) scalability of current approaches to synthesis of Li^+^ ion-selective membranes, (ii) the cost for making large quantities of building blocks of the Li^+^ ion-selective membranes, and (iii) a lack of fundamental understanding of the relationship between Li^+^ ion interactions and dehydration, and the nanochannels’ geometry and chemistry.

A basic understanding of materials’ interactions at the atomic scale, such as their interactions in nanochannels (one or two dimensional), and with negatively charged ligands with conventional membrane matrix materials such as solid-state silicon/AAO and polymers substrates, has not been comprehensively explored. There is a need for research into the easy assembly of Li^+^ ion-selective nanochannels into membrane-like morphologies with high packing density and long-term stability. This research will provide us with information that is critical to the two challenges noted above of scalability and cost.

Most of the proposed Li^+^ ion-selective membranes are synthesized using bottom-up type processes with self-assembly of nanochannels’ reagents, which are challenging to scale up. As a result, the dimensions of the currently proposed Li^+^ ion-selective membranes are very small (mm^2^ scale), and novel methods are required to make these self-assembled building blocks into functional membrane-like morphologies. The cost of raw materials or their processing expense for producing building blocks of Li^+^ ion-selective membranes is currently high. Efficient scale-up and profitable business models for preparing the membranes are required.

Li^+^ ion-selective membranes created with solid-state materials (such as AAO) is an emerging research area. The main challenge for these classes of membranes is their scale-up to practical dimensions for Li^+^ ion extraction. Generating nanoscale channels using current methods such as growing defect-free MOF thin films, aligning carbon nanotubes, or reassembling exfoliated 2D materials, are currently laboratory-scale process. A successful Li^+^ ion-selective membrane requires a high level of nanochannel/nanopore packing in the membrane. In addition, a fundamental challenge still being explored is how to reduce the nanochannel size to near or below the hydration dimeter of Li^+^, without compromising the Li^+^ ions permeation rate. Questions regarding the functional groups that can be used to impart Li^+^ ion discrimination through adjusting the negative charge density of the inner wall surfaces of the channels are also challenges.

It is now clear that the three factors of nanochannel geometry, surface charge density and the nature of materials all play important roles in the design of Li^+^ ion-selective membranes. However, the contribution of each factor, as well as their contributions to Li^+^ ion selectivity and permeability, and membrane stability have yet to be elucidated. Related to this is the question of how to quantify charge density distribution inside nanochannels with a symmetric or asymmetric geometry in order to determine the best materials for Li^+^ ion separation. Currently, there is no assay of sufficient accuracy to quantify the amount of charge inserted per unit length of nanochannel within the membrane.

Although ion transportation mechanisms inside nanochannels have been well-studied, the mechanisms by which Li^+^ ions show higher transportation rates and thus selectivity in some strategically designed nanochannels compared with Na^+^ or K^+^ ions is not yet clear. The observed high Li^+^/Na^+^ or Li^+^/K^+^ selectivities could be attributed to a number of possible reasons, namely different partial dehydration behavior, higher Li^+^ ion mobility after entering the nanochannels, and different affinity of Li^+^ ions to the functional groups inside the nanochannels. This uncertainty and poor understanding, particularly with regards to the dehydration energy barrier, has resulted in contradictory conclusions and discussions. Therefore, more experimental and theoretical exploration is needed to understand what determines Li^+^ ion transportation within the nanochannel at a higher rate than Na^+^ or K^+^.

GO-based Li^+^ ion-selective membranes suffer from a lack of long-term physical and chemical stability, along with the issues of thermal deoxygenation of GO at relatively low temperatures, and low Li^+^ ion permeation rates. The inherent challenge of manufacturing large-scale aligned CNT Li^+^ ion-selective membranes requires novel approaches and more investigative work. Although the recently developed 2D vermiculite membranes show the potential to extract Li^+^ selectively, their poor mechanical strength and brittleness make practical application limited. MOFs, particularly when they are threaded with negative ligands, reveal high Li^+^/Na^+^ or Li^+^/K^+^ selectivities and great potential. However, fabrication of stable defect-free MOF thin film is a significant ongoing issue. Also, producing defect-free MOF thin film on inexpensive, flexible polymeric substrates remains difficult. Creating Li^+^ ion-selective nanochannels on polymeric membranes such as PET using ion etching and UV radiation is currently a lab-scale process and requires expensive infrastructure.

The authors suggest that future efforts should be focused on: (i) improving the fundamental understanding of Li^+^ ion transport mechanisms in nanochannels with extensive theoretical modeling to support experimental results and to guide materials design, (ii) making the Li^+^ ion-selective membrane materials more stable, reversible and durable, (iii) improving Li^+^/Na^+^ or Li^+^/K^+^ selectivities without compromising Li^+^ ion transportation rate, (iv) developing Li^+^ ion-selective thin films on flexible polymeric substrates, and (v) introducing new inexpensive materials that can serve as building blocks for Li^+^ ion-selective membranes. The strong scientific and strategic goals that underpin the need for improved Li^+^ ion separation methods will ensure that this field continues to grow in importance.
